# Remineralization Property of an Orthodontic Primer Containing a Bioactive Glass with Silver and Zinc

**DOI:** 10.3390/ma10111253

**Published:** 2017-10-31

**Authors:** Seung-Min Lee, In-Ryoung Kim, Bong-Soo Park, Dong Joon Lee, Ching-Chang Ko, Woo-Sung Son, Yong-Il Kim

**Affiliations:** 1 Department of Orthodontics, Dental Research Institute, Pusan National University Dental Hospital, Yangsan 50612, Korea; seungminlee@pusan.ac.kr (S.-M.L.); wsson@pusan.ac.kr (W.-S.S.); 2 Department of Oral Anatomy, School of Dentistry, Pusan National University, Yangsan 50612, Korea; biowool@pusan.ac.kr (I.-R.K.); parkbs@pusan.ac.kr (B.-S.P.); 3 Department of Orthodontics, School of Dentistry, University of North Carolina at Chapel Hill, Chapel Hill, NC 27516, USA; DongJoon_Lee@unc.edu (D.J.L.); Ching-Chang_Ko@unc.edu (C.-C.K.); 4 Institute of Translational Dental Sciences, Pusan National University, Busan 46241, Korea

**Keywords:** remineralization, white spot lesion, bioactive glass, silver-doped BAG, zinc-doped BAG

## Abstract

White spot lesions (WSLs) are irreversible damages in orthodontic treatment due to excessive etching or demineralization by microorganisms. In this study, we conducted a mechanical and cell viability test to examine the antibacterial properties of 0.2% and 1% bioactive glass (BAG) and silver-doped and zinc-doped BAGs in a primer and evaluated their clinical applicability to prevent WSLs. The microhardness statistically significantly increased in the adhesive-containing BAG, while the other samples showed no statistically significant difference compared with the control group. The shear bond strength of all samples increased compared with that of the control group. The cell viability of the control and sample groups was similar within 24 h, but decreased slightly over 48 h. All samples showed antibacterial properties. Regarding remineralization property, the group containing 0.2% of the samples showed remineralization properties compared with the control group, but was not statistically significant; further, the group containing 1% of the samples showed a significant difference compared with the control group. Among them, the orthodontic bonding primer containing 1% silver-doped BAG showed the highest remineralization property. The new orthodontic bonding primer used in this study showed an antimicrobial effect, chemical remineralization effect, and WSL prevention as well as clinically applicable properties, both physically and biologically.

## 1. Introduction

White spot lesions (WSLs), which are iatrogenic damages caused by orthodontic treatment, are one of the major causes of esthetic damage. In WSL, the lesions become opaque, making them look whiter than the surroundings owing to the increased porosity of the enamel surface caused by carious demineralization [1]. WSLs are reversible damages that can progress into caries. The incidence of WSL in orthodontic patients varies from 4.9% to 84% and is more prevalent in the upper anterior teeth than in the lower anterior teeth, resulting in an undesirable esthetic state [2,3].

The underlying mechanisms of WSLs due to orthodontic treatment are as follows: First, plaque deposition occurs owing to orthodontic appliance attachment. Because of the orthodontic appliance, a plaque retention area is formed creating an environment where the bacteria can grow easily, and the organic acid generated by the grown bacteria demineralizes the tooth surface. Further, excessive etching to increase the physical adhesion through increased surface areas increases the enamel surface susceptibility [4].

Clinicians have used several methods to prevent this problem because WSLs, which prevail mainly in the anterior teeth, cause esthetic problems. Fluoride application is the most frequently attempted method. Its application in the clinic has the advantage of being applied at a high concentration, but has the disadvantage of increased chair time and costs; further, the frequency of enamel fluoride exposure is low [1]. A fluoride gargle is a method that requires patient cooperation, which has limitations in that the occurrence of WSLs among orthodontic patients is inversely proportional to age [3,5]. If WSLs occur, fluoride application, tooth whitening, porcelain veneers, and other methods are available. However, additional treatment time and costs are incurred, and there is the disadvantage that they do not recover completely [1,2].

To prevent demineralization biochemically, an additive to the bonding system has been studied as a method that does not require patient cooperation. One example is a study attempting to put bioactive glass (BAG) into an adhesive or resin [6]. A BAG has been used in various fields of dentistry, such as for hypersensitivity, remineralization, and antibacterial properties [7]. BAG are the group of glass-ceramic biomaterials. Major component was a SiO_2_ and others were CaO, Na_2_O, P_2_O_5_. BAG can make ion saturation environment by releasing Na^+^, Ca^2+^, PO_4_^3−^, and F^−^ to oral cavity. Near the saturated ions, the calcium phosphate layer grows to apatite and be crystallized. In the oral cavity, sufficient ions can change the demineralization environment to remineralization in balance [8]. A BAG also releases ions to raise the pH to function as a buffer. In other words, it reduces WSLs around the orthodontic bracket owing to the prevention of demineralization from the increased pH and the antibacterial effect [6,9].

Another additive to the bonding system is heavy metals, which have an antibacterial effect. Among them, Ag, which is widely known for its antibacterial effect, was confirmed to inhibit the growth of *Streptococcus mutans* and *Streptococcus sobrinus* in a primer [10]. Ag generates active oxygen to condense bacterial DNA and reduce its replication ability. According to a study by Gargiulo et al., Ag-doped BAG showed a synergistic antibacterial effect [11]. Zinc is another ion with antibacterial and demineralization ability. According to the study by Toledano et al., zinc in dental adhesive showed remineralization effect and Fatima et al.’s study showed antibacterial activity by zinc ion [12,13].

Therefore, the purpose of this study was to find bioactive material with remineralization effect in orthodontic bonding primer. We investigated the possibility of their clinical application by evaluating the mechanical properties, cell viability, remineralization, and antibacterial effect of orthodontic bonding primers containing Ag- or Zn-doped BAG.

## 2. Results

### 2.1. Characteristic of BAG

**Figure 1:**
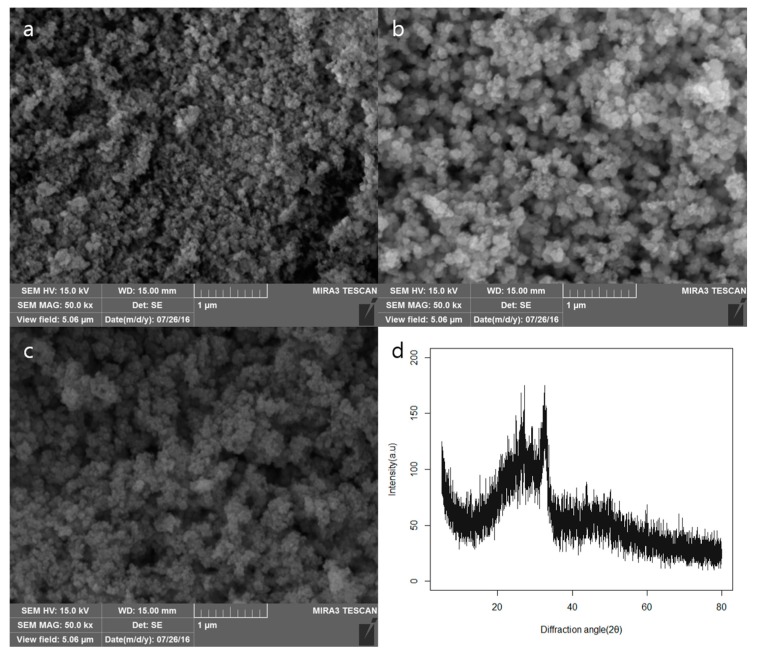
The SEM images of the (**a**) BAG; (**b**) BAG@Ag1 and (**c**) BAG@Zn5; (**d**) XRDpattern of BAG.

In the scanning electron microscope (SEM) image of the heat-treated sample, the polygonal nanoparticles were identified. In this study, the BAG synthesized using the sol-gel synthesis method showed a polygonal particle aggregation similar to those of other studies [14]. The particle size observed in SEM was 100–200 nm. The results showed that there was no diffraction peak except for broad band observed 2θ values in the range of 20°–30°, indicating that the bioactive glass sample showed amorphous properties without a crystalline peak (Figure 1).

### 2.2. Mechanical Properties

#### 2.2.1. Microhardness

**Figure 2:**
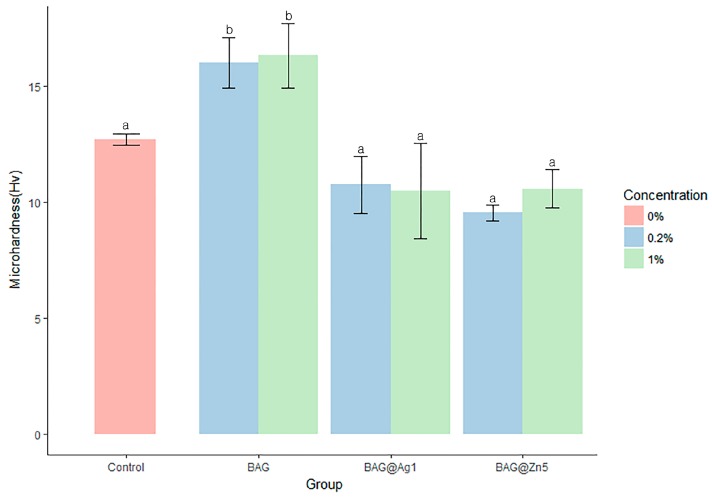
Comparison of the microhardness of the BAG and ion-doped BAG orthodontic bonding primer. Error bars indicate the ± standard deviation. The same letters indicate that the *p*-value is not significantly different (*p* < 0.05). *p* < 0.05 using Duncan’s Multiple Range Test (*n* = 5).

The BAG group was significantly different from the control group; the hardness of the BAG@Ag1 and BAG@Zn5 groups was slightly lower than that of the control group, but showed no statistically significant difference (Figure 2). The BAG, BAG@Ag1, and BAG@Zn5 groups showed no difference in microhardness regardless of the content of the sample added to the orthodontic bonding primer.

#### 2.2.2. Shear Bond Strength

**Figure 3:**
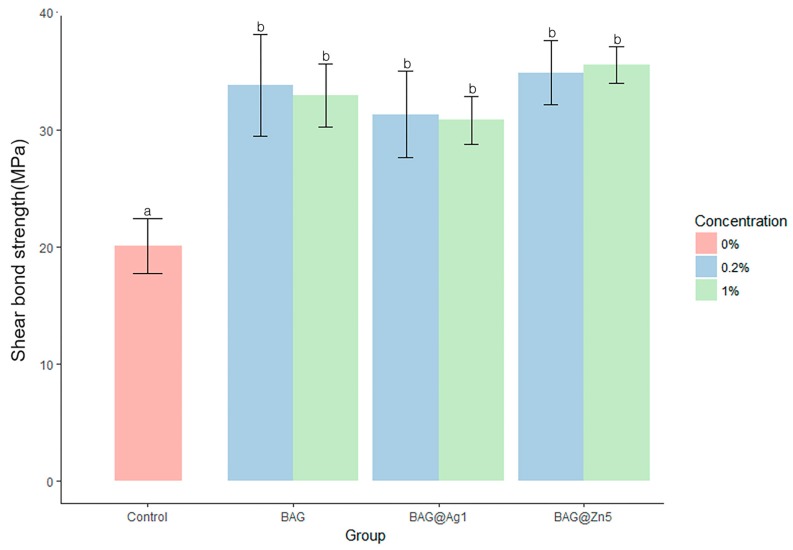
Comparison of the shear bond strength of the BAG and ion-doped BAG orthodontic bonding primer. Error bars indicate the ± standard deviation. The same letters indicate that the *p*-value is not significantly different (*p* < 0.05). *p* < 0.05 using Duncan’s Multiple Range Test (*n* = 5).

**Figure 4:**
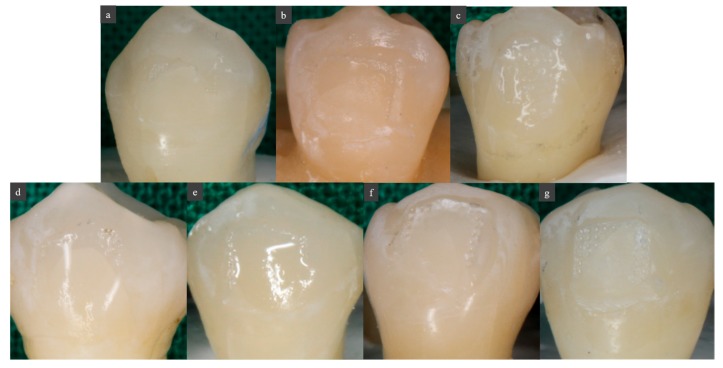
Images of tooth surface after shear bond strength test. (**a**) Control; (**b**) BAG 0.2%; (**c**) BAG 1.0%; (**d**) BAG@Ag1 0.2%; (**e**) BAG@Ag1 1.0%; (**f**) BAG@Zn5 0.2%; (**g**) BAG@Zn5 1.0%.

The sample group showed a significantly higher shear bond strength than the control group. However, there was no statistically significant difference among the BAG, BAG@Ag1, and BAG@Zn5 groups. There was no difference in the shear bond strength according to the sample concentration in the orthodontic bonding primer of the sample (*p* < 0.05) (Figure 3). After shear bond strength test, tooth surface did not shown any other discoloration by ion (Figure 4).

#### 2.2.3. Adhesive Remnant Index (ARI) Score

The 1% sample group showed a less remnant adhesive tendency than the 0.2% sample group; however, no significant difference was observed. There was also no statistically significant difference between the control group and the sample group (*p* < 0.05) (Table 1).

### 2.3. Biological Properties

#### 2.3.1. Cell Viability Test

**Figure 5:**
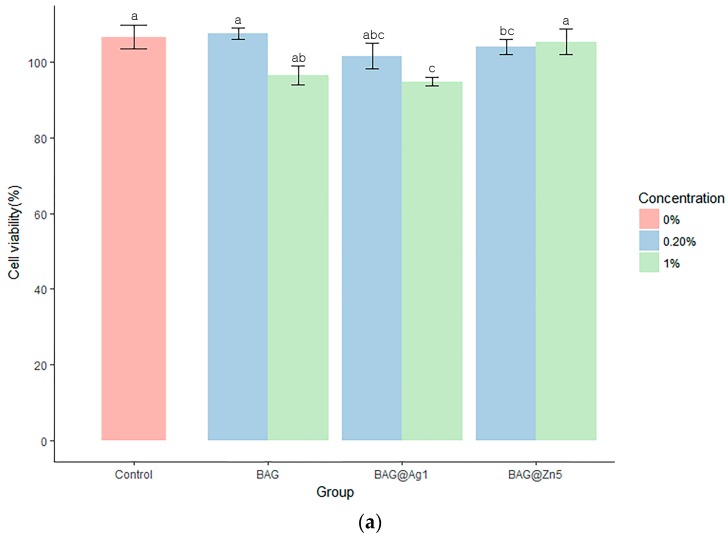

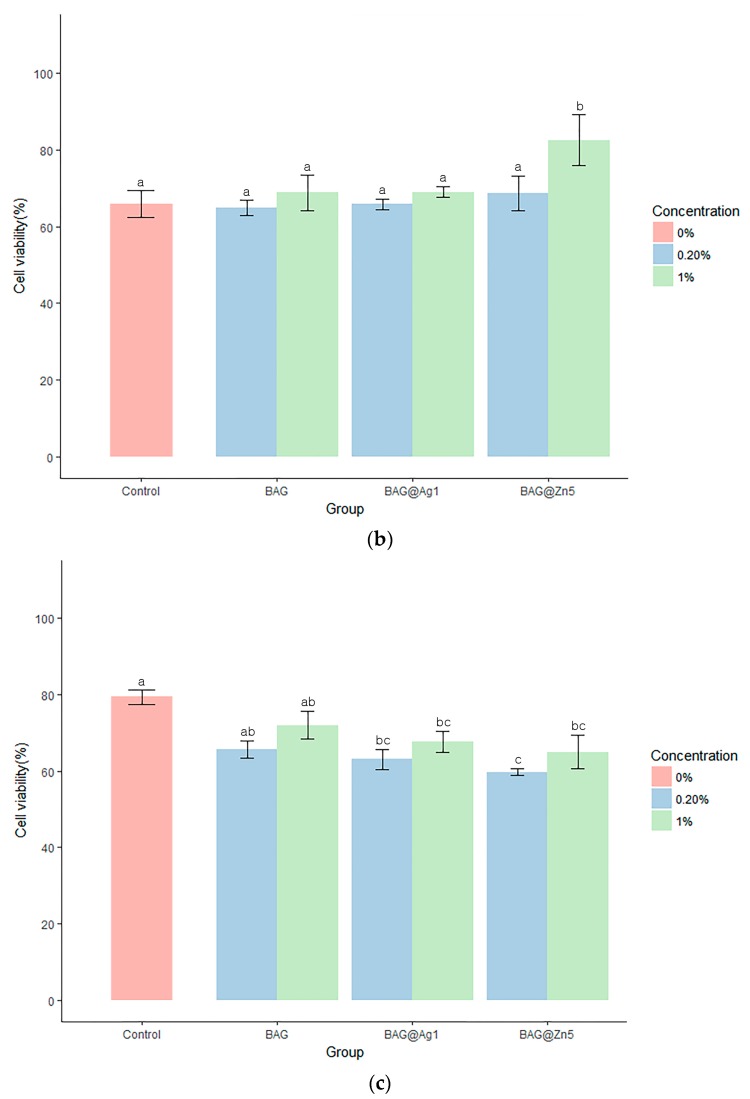
Cell viability test by Human gingival fibroblasts (HGF) cytotoxicity on the cured BAG and ion (silver or zinc)-doped BAG orthodontic bonding primer. (**a**) Cell viability test after 24 h; (**b**) Cell viability test after 48 h; (**c**) Cell viability test after 72 h. Viability is measured using MTT((3-(4,5-Dimethylthiazol-2-yl)-2,5-Diphenyltetrazolium Bromide) absorbance on 620 nm. Duncan’s Multiple Range Test (*n* = 3). The same letters indicate that the *p*-value is not significantly different (*p* < 0.05). Error bars indicate the ± standard deviation.

The cell viability after 24 h was significantly lower in the BAG@Ag1 group than in the control group. The comparison of the concentration differences among the groups showed that the higher the concentration in BAG and BAG@Ag1, the lower the cell viability was. After 48 h, the cell viability was significantly higher only in the group containing 1% of BAG@Zn5. After 72 h, it was significantly lower in the sample group than in the control group, and there was no difference among the sample groups. The comparison of the concentration differences among the groups showed that the cell viability was higher in the 1% group than in the 0.2% group (Figure 5).

#### 2.3.2. Antibacterial Properties

Regarding antibacterial activity, there was a significant difference between the group containing the sample in the orthodontic bonding primer and the negative control with no additive (*p* < 0.05) (Table 2).

### 2.4. Remineralization Test

**Figure 6:**
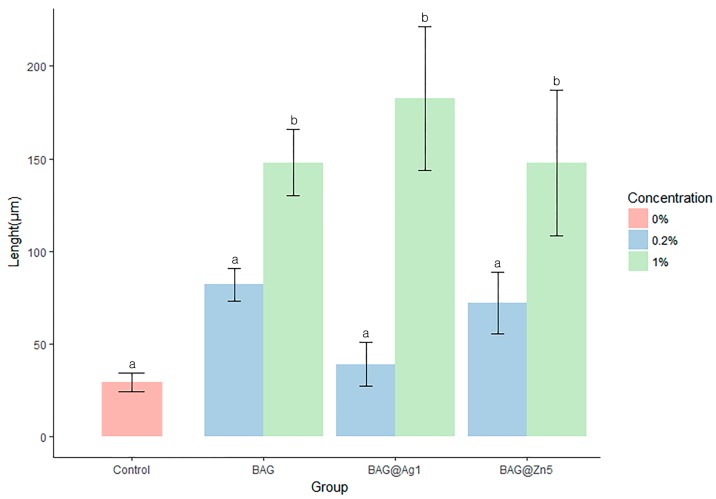
Comparison remineralization length of the BAG and ion (silver or zinc)-doped BAG orthodontic bonding primer using the ImageJ analysis. Duncan’s Multiple Range Test (*n* = 9). The same letters indicate that the *p*-values are not significantly different (*p* < 0.05). Error bars indicate the ± standard deviation.

**Figure 7:**
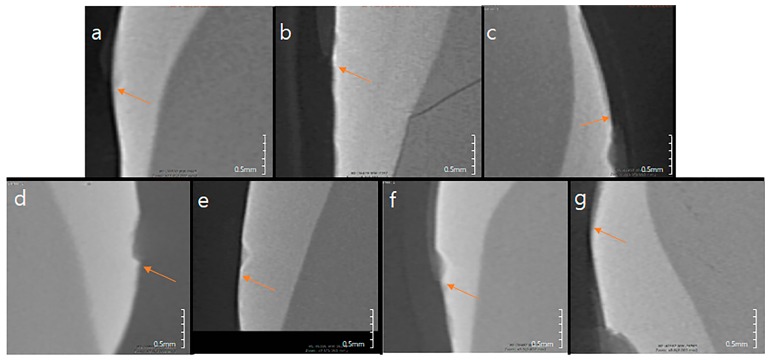
Remineralization point of the BAG and ion (silver or zinc)-doped BAG orthodontic bonding primer via CBCT. (**a**) Control; (**b**) BAG 0.2%; (**c**) BAG 1.0%; (**d**) BAG@Ag1 0.2%; (**e**) BAG@Ag1 1.0%; (**f**) BAG@Zn5 0.2%; and (**g**) BAG@Zn5 1.0%.

The remineralization results showed that the 0.2% BAG, BAG@Ag1, and BAG@Zn5 groups showed no significant difference compared with the control group; however, the 1% BAG, BAG@Ag1, and BAG@Zn5 groups showed a significant difference compared with the control group (Figure 6 and Figure 7). In other words, the remineralization properties in each group were noted to be concentration-dependent compared with that in the control group. Among the samples, the orthodontic bonding primer containing 1% of BAG@Ag1 showed the highest remineralization property.

## 3. Discussion

WSLs are one of the side effects of orthodontic treatment that can cause esthetic problems for patients. These can be managed with fluoride application, restorative treatment, or prosthetic treatment; however, complete recovery is difficult owing to irreversible phenomena [2,3]. Therefore, prevention is important before such phenomena occur. To prevent WSLs, oral hygiene control through oral hygiene education of patients is important; however, it is difficult to obtain patient cooperation in young orthodontic patients. Owing to these problems, attempts have been made to introduce a biological or chemical agent into the bonding agent. Therefore, studies have been conducted on the addition of BAG or metal ions to bonding agents.

This study examined the possibility of WSL prevention using antimicrobial ability and remineralization testing by mixing BAG- and Zn-doped BAGs with orthodontic adhesive and evaluated the possibility of their clinical application as bonding agents via assessment of their properties and toxicities.

In the evaluation of physical properties, the microhardness of the orthodontic bonding primer with BAG increased, and that of the BAG@Ag1 and BAG@Zn5 was not significantly different from that of the control group. This is consistent with the findings of a previous study that showed an increased microhardness owing to a filler insertion effect by adding BAG in the resin paste [9].

The shear bond strength increased in all samples compared with that in the control group. However, the orthodontic adhesive containing Ag in the study by Ahn et al. did not show any difference in shear bond strength and ARI score between the nanoparticle and microparticle Ag [15]. Compared with other previous studies where the shear bond strength decreased or showed no changes, the shear bond strength increased in this study [9,16,17,18]. This is because the adhesive used in the study by Ahn et al. contained 0.025% or 0.05% of Ag, which increased the roughness, but not enough to increase the frictional force [15]. In this study, the content in the BAG, BAG@Ag1, and BAG@Zn5 groups was 0.2% and 1%, which suggests that the irregularity of the surface increased to increase the bonding strength as well.

Regarding cell viability, there was no difference between the control group and orthodontic bonding primer containing BAG and ion-doped BAG groups after 24 h. However, the viability decreased in the experimental group after 48 and 72 h. This is similar to the findings of a previous study showing a slight decrease in cell viability over time [6]. The cytotoxicity of the adhesive was due to an unreacted bisphenol A, and the amount of non-polymerized bisphenol A was reported to be very small [19].

All samples showed statistically significant antibacterial properties compared to the sterilized saline. A BAG reduces bacterial metabolism by decreasing the level of PO_4_. Its antibacterial effect reduces the occurrence of WSLs by blocking enamel demineralization [20]. The antibacterial effect of Ag originates from the Ag ion repositioning from molecules to produce active oxygen causing structural damage of bacteria [15]. Previous studies have shown antibacterial effects on *S. mutans* in Ag-added bonding agents. *S. mutans* is a contributor to caries in oral environments and increases in orthodontic treatment [21]. Ag showed an antibacterial effect at 50 µg/mL [4,21]. The flowable resins containing 1–5% Zn showed an antibacterial effect on *S. mutans*. Among these samples, 1% Zn showed an antibacterial effect with proper mechanical properties for clinical application such as cure depth, flexural strength and modulus, and compressive strength and modulus [22]. The remineralization tests showed the effect of remineralization in a concentration-dependent manner. The group containing 0.2% of each sample showed a remineralization effect compared with the control group; however, it was not statistically significant. However, all groups containing 1% samples showed a statistically significant remineralization effect compared with the control group. As a result of remineralization test, the longest remineralization length among the sample was the orthodontic bonding adhesive containing 1% BAG@Ag1. A BAG is a silica-based ion releasing agent and has been reported to have a buffering effect to raise the pH lowered by bacteria [9]. In the resin paste containing BAG, the occurrence of WSLs around the orthodontic bracket was prevented, and the enamel hardness was maintained [23]. The addition of 25–50% of BAG to the orthodontic bonding agent showed an ion release and provided an ion source for the tooth surface as a reservoir of Ca and PO_4_. In addition, Zn forms an α-hopeite-like phase in the PO_3_, which is the site of the hydroxyapatite of the enamel surface. The enamel surface containing Zn enters a low solubility state and can withstand acidic conditions [24,25,26].

In the abovementioned study, the surface application of more than 1% of the adhesive was difficult owing to the increased viscosity, which is the nature of the bonding agent, although the sample placed in the adhesive showed a remineralization effect in a concentration-dependent manner. A limitation of the addition of Ag in an orthodontic bonding primer is that a yellow/brown color appears due to the oxidation of Ag when it contains more than 2% [6]. In this study, 1% Ag-containing orthodontic bonding primer was clinically acceptable. In this study, the Ag content of 0.01% and 0.002% was lower than those of previous studies involving Ag contents of 0.11%, 0.18%, and 0.33%. The yellow/brown color was also at clinically acceptable levels [16,18]. The concentration of Zn was 0.01%, which was 0.5% lower than 1–5% in a previous study [22]. In this study, an effect on WSLs was expected owing to the antibacterial effect and ion capacity when orthodontic bonding primers containing low concentrated BAG, Ag, and Zn were used clinically. The biological and mechanical properties are considered to be at a clinically acceptable level. In addition, it can be applied to susceptible enamel surfaces by surplus etching to lower the possibility of WSL occurrence. However, no oral environment mimic test was performed in this study. Because more than 1 year of orthodontic appliance attachment is often necessary in orthodontic treatment, a follow-up study is needed to determine the duration of the sample affecting the enamel surface.

## 4. Materials and Methods

### 4.1. Synthesis of Ag- and Zn-Doped BAGs

According to the quick alkali-mediated sol-gel method proposed by Xia and Chang, the materials were compounded in the same ratio as in Table 3 [27].

The synthesis of BAG was as follows. Step 1: 2.8 mL of 2 M NHO_3_ (Samchun, Seoul, Korea) and 13.9 mL of distilled water (Samchun, Seoul, Korea) were added to 50 mL of ethanol and stirred at 25 °C for 30 min at 600 rpm to prepare an aqueous acid solution. Thereafter, 21.6 mL of tetraethyl orthosilicate (Sigma-Aldrich, St. Louis, MO, USA) was added to the prepared aqueous acid solution and stirred at room temperature for 30 min; 2.2 mL (12.95 mmol) of triethyl phosphate was then added, and the mixture was stirred for another 30 min. Step 2: In step 1 solution, 14.04 g of Ca(NO_3_)_2_·4H_2_O were added, and the mixture was stirred for 30 min to create a clear sol state. Step 3: In the prepared sol, 10 mL of 2 M NH_4_OH (Samchun, Seoul, Korea) was added and stirred for 30 min to allow the sol to form a gel. The obtained gel was stirred using a muddler to prevent bulking of the gel. The resulting gel was aged in a 60 °C-oven for 24 h. Dry gel powder was heat treated in a 600 °C-furnace for 4 h.

For BAG@Ag1, Step 1 was same process. Step 2: In step 1 solution, 13.5 g of Ca(NO_3_)_2_·4H_2_O were added, and the mixture was stirred for 30 min to create a clear sol state. At that time, 1.82 g (10.7 mmol) of AgNO_3_ were added to the solution. Step 3 was also same process.

For BAG@Zn5, Process of step 1 was same. Step 2: In step 1 solution, 11.91 g of Ca(NO_3_)_2_·4H_2_O were added, and the mixture was stirred for 30 min to create a clear sol state. At that time, 0.15 g (0.5 mmol) of Zn(NO_3_)_2_·6H_2_O were added to the solution. Step 3 was also same process.

### 4.2. Characterization of the Nano-BAGs

The shape of the sample was analyzed using field-emission scanning electron microscopy (SUPRA25; Carl Zeiss, Oberkochen, Germany). *x*-ray diffraction (XRD) patterns were acquired by an Ultima IV multipurpose XRD system (Rigaku, The Woodland, TX, USA) at 40 kV and 40 mA, with a scanning speed of 0.1°/min.

### 4.3. Mechanical Properties

#### 4.3.1. Disk Preparation for Mechanical Properties

A disk (diameter, 5 mm; height, 1 mm) was created to evaluate the properties of the orthodontic bonding primers containing ion-doped BAG. To prevent transmission of light, 1 mL of orthodontic bonding primer (Transbond™ XT Primer3M, Monrovia, CA, USA) was filled in black e-tube. 1 or 0.2 wt % of each BAG, BAG@Ag1, and BAG@Zn5 put in the black e-tube and then shaken using a mixer twice for 20 s. A homogeneously mixed sample was injected to the brass mold, the glass microscope slide (thickness: 0.2 mm) was covered on the top for making flat surface. Light cured using VALO (Ultradent Products, South Jordan, UT, USA) for 20 s. All sample ion concentration was based on previous studies and aim was finding the minimum concentration with antibacterial and remineralizaiton effect [6,10,22] (Table 4).

#### 4.3.2. Microhardness

Five disks in each group were loaded with 200 gf to measure the hardness Vickers using a microhardness tester (MVK-H1, Akashi, Japan).

#### 4.3.3. Shear Bond Strength

A total of 35 premolars that were extracted for the purpose of orthodontic treatment were prepared for each group (five each). This study was reviewed and approved by the Institutional Review Board of Pusan National University Dental Hospital (PNUDH-2016-025). The teeth with enamel defects, such as WSLs, including caries were excluded. The surface to which the bracket to be attached was cleaned using a prophylaxis cup and fluorine-free pumice, rinsed for 10 s, and dried. It was etched using 35% phosphoric acid gel (Ultra Etch, Ultradent, South Jordan, UT, USA) for 15 s, suctioned, rinsed, and dried. On the dried surface, the sample was applied and gently aired for 2 s. The Transbond™ XT light cure adhesive paste was applied to the premolar bracket (Arista™, Select Dental, Farmingdale, NY, USA) and attached parallel to the major axis of the tooth, and the remaining paste was removed and light cured for 5 s at the medial and distal sides. All of these procedures were performed in accordance with the recommended instructions for the Transbond™ XT Primer. The bracket-bonded teeth were stored in distilled water for 24 h and then measured using a universal testing machine (3300 Universal Testing Systems, Instron Corporation, Canton, MA, USA). The maximum load (N) was measured at a crosshead speed of 1 mm/min after the steel rod of the machine was placed vertically on the bracket. The measured load (N) value was divided by the bracket base area (11.83 mm^2^) and converted to bond strength (MPa). The bonding failure of debonding of the tooth surface was evaluated using the ARI score. The evaluation criteria were as follows: 1—All the adhesive remained on the tooth; 2—More than 90% of the adhesive remained on the tooth; 3—Between 10% and 90% of the adhesive remained on the tooth; 4—Less than 10% of the adhesive remained on the tooth; and 5—No adhesive remained on the tooth.

### 4.4. Biological Properties

#### 4.4.1. Cell Viability Assay

The disk was disinfected with EO gas, placed in a 96-well plate, and UV exposed for 100 min. Human gingival fibroblasts (HGF-1 (ATCC, Rockville, MD, USA)) and 10% fetal bovine serum (FBS, Hyclone Logan, UT, USA) were cultured in Dulbecco’s modified Eagle’s medium (Hyclone Logan, UT, USA) containing 10% fetal bovine serum and 100 IU/mL penicillin/streptomycin (Hyclone Logan, UT, USA). HGF-1 cells were dispensed in 96-well plates and incubated for 24 h in a 37 °C-5% CO_2_ incubator. After incubation, MTT [3-(4,5-dimethylthiazol-2-yl)-2,5-diphenyltetrazolium bromide] (Sigma-Aldrich, St. Louis, MO, USA) was added at a concentration of 5 mg/mL and reacted for 4 h in a dark room. Thereafter, the supernatant was removed and dissolved in MTT crystal dimethyl sulfoxide (Sigma-Aldrich, USA, 150 µL/well) formed in the cells, and the absorbance (Sunrise™, TECAN, Männedorf, Switzerland) at 620 nm was measured.

#### 4.4.2. Antibacterial Test

The *S. mutans* (KFCC, Seoul, Korea) used in this study was cultured in a brain heart infusion medium in a 37 °C-incubator. The disk was sterilized by EO gas, placed in a 96-well plate, and UV exposed for 100 min. Thereafter, *S. mutans* (1.0 × 105 CFU/mL) was added and incubated in a 37 °C incubator for 24 h and 48 h. The absorbance (Sunrise™, TECAN, Männedorf, Switzerland) was then measured at 620 nm.

### 4.5. Remineralization Test

**Figure 8:**
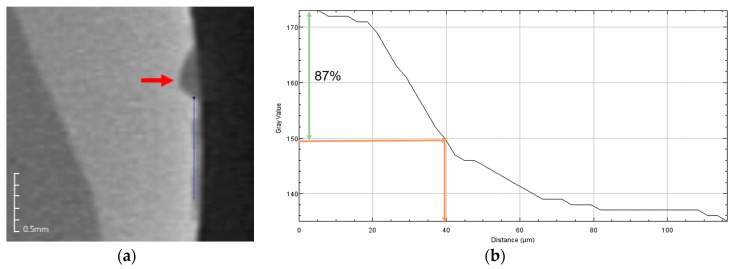
Remineralization length analysis method. (**a**) Micro CT (computer tomography) slice of the region of interest at the center of the lesion perpendicular to the enamel surface, red arrow: Perforated landmark for a reference point, blue line: line of interest region from reference point on enamel surface; (**b**) Histogram in ImageJ. Green arrow: up to 87% level of gray value from the reference point, orange arrow: the distance at the 87% gray value from reference point.

The pH cycling method proposed by Toda and Featherstone was used to examine the remineralization effect [28]. Among the premolars extracted for the purpose of orthodontic treatment, those without WSLs, caries, or enamel defects were divided into groups, nine for each group. Each tooth was embedded in acrylic resin (Caulk Orthodontic Resin, Dentsply Caulk, York, PA, USA) using a mold. The tooth surface to be bonded with the embedded tooth sample was cleaned using fluorine-free pumice and prophylaxis cup, rinsed for 10 s, and dried. The cellophane tape was attached so that the surface, except the tooth surface of 5 mm × 5 mm, was not etched, and the corners were perforated using a dental bur so that the boundary could be found easily. The exposed tooth surface was etched with 35% phosphoric acid gel (Ultra-etch, Ultradent, South Jordan, UT, USA) for 30 s, rinsed for 10 s, and dried. Using the same procedure for the disk, the sample and the orthodontic bonding primer were mixed and applied to the tooth surface and light cured for 5 s, and the cellophane tape was removed. The teeth were stored in distilled water for 24 h and placed in a demineralizing solution (Biosesang, Seongnam-si, Korea) for 6 h and a remineralizing solution (Biosesang, Korea) for 18 h (Table 5). This cycle was repeated for 14 days. The solution was replaced with a new one weekly. The solution was washed with distilled water for 1 min between the demineralizing solution and remineralizing solution daily, dried with gentle air, and replaced with a solution. Micro-CT (InspeXio SMX-90CT Plus Benchtop Micro Focus *x*-ray, Shimadzu, Kyoto, Japan) was used at 90 KV and 109 µA for the measurement. The measured micro-CT data were analyzed using ImageJ (National Institutes of Health, Bethesda, MD, USA) (Figure 8) [29]. On the ImageJ, the length was corrected to the scale bar on the micro-CT. Using the histogram of brightness, the sound enamel was set up to 87% contrast, and the distance from the final point where the sample orthodontic bonding primer was applied was measured to determine the remineralization length. We calibrated the scale on the micro-CT slice using the function of set scale on ImageJ. After turning on the Histograms’ live function, we chose the region of interest on enamel surface by freehand line. We determined 87% gray value from reference point, and then the distance of 87% gray value from reference point was defined as the remineralization length.

### 4.6. Statistical Analysis

The one-way analysis of variance was used to compare microhardness, shear bond strength, antibacterial test results, cell viability test results, and pH cycle between groups, and Duncan’s new Multiple Range Test was used for post-testing. The Kruskal-Wallis test was performed for the ARI score. All statistical analyses were performed using the R language program (version 3.3.3; R Foundation for Statistical Computing, Vienna, Austria).

## 5. Conclusions

The following results were obtained in this study. The BAG and Ag- or Zn-doped BAG orthodontic bonding primers showed antibacterial and remineralization properties. Thus, the sample group had a capacity for preventing WSLs. Among the samples, the Ag-doped BAG showed the highest remineralization property. The BAG and Ag- or Zn-doped BAG orthodontic bonding primers were mechanically and biologically acceptable to be clinically applied in patients.

## Figures and Tables

**Table 1:** Adhesive Remnant Index (ARI) scores of orthodontic bonding agents tested.

ARI (%)	Control	BAG		BAG@Ag1		BAG@Zn5		Significant
		0.2%	0.5%	0.2%	0.5%	0.2%	0.5%	
1	0	0	0	0	0	0	0	Not significant
2	3	2	2	0	2	0	2	
3	1	2	2	5	3	5	3	
4	1	1	1	0	0	0	0	
5	0	0	0	0	0	0	0	

The ARI score is not significantly different according to the Kruskal-Wallis test at α = 0.05 (*n* = 5); Score 1—All the adhesive remained on the tooth; Score 2—More than 90% of the adhesive remained on the tooth; Score 3—Between 10% and 90% of the adhesive remained on the tooth; Score 4—Less than 10% of the adhesive remained on the tooth; Score 5—No adhesive remained on the tooth.

**Table 2:** Antibacterial properties difference between the BAG and ion (silver or zinc)-doped BAG orthodontic bonding primer.

Group		24 h				48 h				Significance
		Optical Density (Absorbance at 650 nm)				Optical Density (Absorbance at 650 nm)				
Negative control ^†^ (sterile saline)		0.28	±	0.02	a	0.78	±	0.07	a	*p* < 0.001 ^‡^
BAG	0.2%	0.01	±	0.00	b	0.01	±	0.00	b	
	1%	0.01	±	0.00	b	0.01	±	0.00	b	
BAG@Ag1	0.2%	0.01	±	0.00	b	0.02	±	0.01	b	
	1%	0.01	±	0.00	b	0.04	±	0.02	b	
BAG@Zn5	0.2%	0.01	±	0.01	b	0.02	±	0.01	b	
	1%	0.01	±	0.00	b	0.01	±	0.00	b	

^†^ Negative control means that it contains nothing; ^‡^ Duncan’s Multiple Range Test at *p* < 0.05 (*n* = 3).

**Table 3:** Composition of the ion-doped bioactive glass.

Group	Composition (wt %)				
	SiO_2_	CaO	P_2_O_5_	Ag_2_O	ZnO
BAG	58	33	9	0	0
BAG@Ag1	58	32	9	1	0
BAG@Zn5	58	28	9	0	5

**Table 4:** Sample group in this study.

Group		Composition (wt %) in Orthodontic Bonding Primer		
		BAG	Silver	Zinc
Control		0	0	0
BAG	0.2%	0.200	0	0
	1.0%	1.000	0	0
BAG@Ag1	0.2%	0.198	0.002	0
	1.0%	0.990	0.010	0
BAG@Zn5	0.2%	0.190	0	0.010
	1.0%	0.950	0	0.500

**Table 5:** Composition of the remineralizing and demineralizing solutions.

Solution	Composition	
Demineralizing solution (pH 4.4)	Calcium 2.0 mmol/L	Ca(NO_3_)_2_·4H_2_O
	Phosphate 2.0 mmol/L	KH_2_PO_4_
	Acetic acid 75.0 mmol/L	CH_3_COOH
Remineralizing solution (pH 7.0)	Calcium 1.5 mmol/L	Ca(NO_3_)_2_·4H_2_O
	Phosphate 0.9 mmol/L	KH_2_PO_4_
	KCl 130.0 mmol/L	KCl
	Sodium cacodylate 20.2 mmol/L	NaC_2_H_6_AsO_2_·3H_2_O
